# Circulatory Levels of RANKL, OPG, and Oxidative Stress Markers in Postmenopausal Women With Normal or Low Bone Mineral Density

**DOI:** 10.1177/1177271919843825

**Published:** 2019-08-19

**Authors:** Fawaz Y Azizieh, Diaa Shehab, Khaled Al Jarallah, Renu Gupta, Raj Raghupathy

**Affiliations:** 1 Department of Mathematics and Natural Sciences, International Centre for Applied Mathematics and Computational Bioengineering, Gulf University for Science and Technology, Hawally, Kuwait; 2 Department of Medicine, Faculty of Medicine, Kuwait University, Kuwait, Kuwait; 3 Department of Radiology, Faculty of Medicine, Kuwait University, Kuwait, Kuwait; 4 Department of Microbiology, Faculty of Medicine, Kuwait University, Kuwait, Kuwait

**Keywords:** circulatory levels, RANKL, OPG, oxidative stress, postmenopausal, BMD osteoporosis

## Abstract

**Introduction:**

Receptor activator of nuclear factor κB ligand (RANKL), osteoprotegerin (OPG), and oxidative stress markers are suggested to contribute to bone loss in osteoporosis that occurs in menopause. However, the association between these markers and bone mineral density (BMD) is controversial. The aim of this study was to measure circulatory levels of these parameters in postmenopausal women with normal or low BMD.

**Methods:**

The study population included 71 postmenopausal women, of whom 25 had normal BMD, 31 had osteopenia, and 15 had osteoporosis. Serum levels of RANKL, OPG, and 5 oxidative stress markers (catalase, peroxiredoxin 2 [PRX2], superoxide dismutase 1 [SOD1], superoxide dismutase 2 [SOD2], and thioredoxin [TRx1]) were measured using the Multiplex system.

**Results:**

As compared with subjects having normal BMD, subjects with low BMD had significantly lower median serum levels of OPG, catalase, SOD2, and PRX2 (*P* = .004, .031, .044, and .041 respectively). Although levels of RANKL were not different between the 2 groups, the RANKL/OPG ratio was higher in women with low BMD (*P* = .027).

**Conclusions:**

These data provide insights into the possible roles of OPG, RANKL, and oxidative stress in the pathogenesis of postmenopausal osteoporosis. However, the lack of association between these markers and BMD indicates that osteoporosis is complex and multivariate.

## Introduction

Osteoporosis continues to be a serious health issue associated with aging.^1^ With the increase in life expectancy, osteoporosis is becoming a major health problem worldwide. Clinically, osteoporosis is a skeletal disorder characterized by compromised bone strength, which predisposes a person to an increased risk of fracture. On the cellular level, research has focused on factors that help reduce the activity of osteoclasts and/or increase the activity of osteoblasts, thus reducing bone turnover. This has included research on elucidating the pathophysiology of osteoporosis, as well as monitoring, modulating, controlling, and reversing the osteoporotic processes. This study is an effort along these lines, aimed at furthering our understanding of the association between several circulatory markers and postmenopausal osteoporosis.

Postmenopausal osteoporosis continuous to pose a significant challenge partially due to the great deal of controversy related to contributory factors and markers. In this context, estrogen is the most extensively and intensively investigated factor considering that its levels decline with the onset of menopause. An impressive number of studies indicate a fundamental role for estrogen in maintaining bone density.^2–4^ Estrogen reduces burn turnover by suppressing the differentiation of osteoclasts, enhancing the apoptosis of osteoclasts, enhancing the differentiation of osteoblasts, and increasing their life span by decreasing apoptosis.^2,3^ Whereas these effects are at least in part mediated directly through high-affinity estrogen receptors on bone cells, it is now becoming clear that much of it is through several other pathways. Estrogen suppresses the production of proresorptive cytokines such as tumor necrosis factor-alpha (TNF-α), interleukin (IL)-1, IL-8, IL-6, IL-17 as well as the receptor activator of nuclear factor κB ligand (RANKL); on the contrary, it increases the production of anti-resorptive cytokines such as transforming growth factor-beta (TGF-β), interferon-gamma (IFN-γ) as well as osteoprotegerin (OPG).^5–8^

RANKL promotes the differentiation and activation of osteoclasts and stimulates and maintains their resorption activity.^9,10^ It has also been proposed that many osteoporotic pathways, such as those induced by cytokines or hormones, are primarily mediated by inducing RANKL expression in osteoblast lineage cells.^7,8^ Studies on mice revealed that the administration of soluble RANKL results in an increase in the formation and activation of osteoclasts that lead to osteoporosis.^11^ On the other hand, OPG is a potent inhibitor of osteoclast formation and acts as a decoy receptor for RANKL. In vivo experiments showed that OPG knockout mice develop severe osteoporosis, whereas treatment of normal mice with OPG leads to osteopetrosis.^12^ Research on RANKL and OPG has highlighted their contribution to molecular processes in osteoporosis, promoting the idea of using them as targets for management.^10,13–16^

Oxidative stress (OS) occurs as a result of an imbalance between the production of reactive oxygen species (ROS) and the removal of their reactive intermediates. ROS are products of the oxidative phosphorylation pathway in mitochondria and are chemically reactive oxygen molecules that include hydrogen peroxide, nitric oxide, superoxide, and other hydroxyl radicals. ROS is associated with OS which suggests that ROS induce pathology by damaging lipids, proteins, and DNA.^17^ In response to ROS-mediated damage, cells defend themselves by clearing ROS molecules through enzymes such as superoxide dismutases (SODs), catalases, glutathione peroxidases, and peroxiredoxins. In normal conditions, catalase, SOD1, and SOD2 rapidly convert the superoxide and hydrogen peroxide into oxygen and water to minimize the damage to cells. SOD1 is primarily located in the cytosol and mitochondrial intermembrane space, whereas SOD2 is located in the mitochondrial matrix. SODs prevent accumulation of superoxide that can damage and inactivate proteins containing iron-sulfur clusters.^18^

However, when the ROS clearance pathway is impaired, accumulation of ROS can cause OS to the cells and induce DNA damage, lipid peroxidation, and enzyme inactivation. On the other hand, ROS can be beneficial, as when they are used by the immune system to attack and kill pathogens. Furthermore, OS or the maintenance of a physiological level of oxidant challenge is essential for governing life processes through redox signaling. Thus, the ability to monitor the level of OS and the balance between ROS production and clearance within cells is key to the understanding of many diseases.^19,20^

OS has been reported to be involved in several acute and chronic diseases and pathological conditions including osteoporosis.^19,21–23^ ROS are known to have several effects on bone cells (reviewed in Abdollahi et al^24^). OS has inhibitory effects on osteoblasts, including the inhibition of proliferation and differentiation, inhibition of mineralization, and induction of necrosis. These effects have been shown to be counteracted by antioxidants.^25^ On the other hand, ROS such as superoxide and hydrogen peroxide have been shown to regulate osteoclastic bone resorption. This includes enhancement of the activity, proliferation, and differentiation of osteoclasts. Furthermore, superoxides generated from osteoclasts directly contribute to bone degradation, whereas the inhibition of osteoclastic superoxide causes a reduction in bone resorption.^26^ OS also causes partial degradation of fibronectin, which is a major component of the extracellular bone matrix and is involved in the adhesion, proliferation, migration, and differentiation of osteoblasts.^24,27^

This study was aimed at measuring circularity levels of RANKL, OPG, and OS markers in postmenopausal women with normal and low bone mineral density (BMD).

## Methodology

### Patient selection

This study focused on 71 women who were postmenopausal (ie, absence of menstrual periods for at least 12 months before the study). Participants were recruited from the Physical Medicine Unit at Mubarak Al Kabeer Hospital, Kuwait; they were clinically assessed by a single physician. Of these women, 25 had normal BMD and 46 had low BMD. Participants were categorized further into 3 groups based on *T*-scores of BMD: the normal group (N; *T*-scores ⩾ –1, n = 25), the osteopenia group (OSN; –2.5 < *T*-scores < –1, n = 31), and the osteoporosis group (OSR; *T*-scores ⩽ –2.5, n = 15). This was based on the guidelines set by the World Health Organization (WHO) and Adult Official Positions of the International Society for Clinical Densitometry (ISCD; http://www.iscd.org/official-positions/2015-iscd-official-positions-adult/) updated in 2015. Demographic data such as age, weight, height, body mass index (BMI), and duration since menopause were recorded on the day of examination. Women who were on systemic corticosteroids or who had malignancy, hyperparathyroidism, severe renal impairment, liver disease, or experiencing any infectious disease were excluded. Women who needed calcium and vitamin D supplementation were receiving 600 mg of calcium and 200 IU of vitamin D twice daily. Women with normal BMD or osteopenia were not on any bone active agents, whereas women with osteoporosis were taking bisphosphonates. This study received ethical approval from the Ethics Committee of the Health Sciences Center of Kuwait University, and participants gave written informed consent prior to participating in the study.

### BMD measurement

Total body BMD and bone mineral content (BMC) were measured using dual-energy X-ray absorptiometry (GE Medical System Lunar, Madison, WI, USA). Total BMD and BMC were measured with a precision (coefficient of variation) of 0.7%. BMD was measured at total lumbar spine (L1-L4) and left hip. The diagnosis was done by a single consultant using *T*-score values according to the criteria set by WHO as mentioned above.

### Blood sampling and sample storage

Venous blood samples were collected in vacutainer tubes and allowed to clot at room temperature for 30 minutes. The coagulated blood was centrifuged for 10 minutes at 3000×*g*; serum samples were aliquoted into sterile tubes and stored frozen till analysis.

### Estimation of circulatory levels of RANKL, OPG, and OS markers in serum samples

A multiplex enzyme-linked immunosorbent assay (ELISA), containing dyed microspheres conjugated with target-specific monoclonal antibodies, was used according to the manufacturer’s (Merck Millipore, Darmstadt, Germany) instructions.

Serum levels of bone markers were measured using commercially available multiplex ELISA (MILLIPLEX MAP; Merck Millipore) to measure RANKL (HRNKLMAG-51K) and OPG (HBNMAG-51K). The minimum detectable concentrations of these assays were 5.0 and 1.9 pg/mL, respectively.

Serum levels of OS parameters were measured using the Human Oxidative Stress Magnetic Bead Panel (H0XSTMAG-18K; Merck Millipore). The panel tested for catalase, peroxiredoxin 2 (PRX2), SOD1, SOD2, and thioredoxin (TRX1). The multiplex system allowed the simultaneous measurement of the median fluorescence intensity (MFI) for all the parameters in each reaction well.

Serum levels of all analytes were determined using MagPix Array Reader (Luminex Manager Software). Quality control measures were followed as per the recommendations of the manufacturer. The intra-assay and inter-assay coefficients of variation (CVs) for all parameters tested were <10% and <15%, respectively.

### Statistical analysis

Statistical analysis was performed using the SPSS version 23 software (SPSS Inc, Chicago, IL, USA). Normality distribution of data was first determined by the Kolmogorov-Smirnov test, and groups were accordingly compared using one-way analysis of variance (ANOVA), Student *t*-test, or Mann-Whitney *U* test. Categorical variables were compared using Pearson chi-square test. Spearman rank correlation coefficient was calculated to determine correlations between different measures and cytokine levels. A *P*-value less than or equal to .05 was considered statistically significant for all tests.

We further used regression models to ascertain whether the age, years since menopause, and BMI were potential confounders in the search for possible associations between BMD groups and different markers, particularly as some of these variables were different between the 2 groups. Given the binary outcome variable (2 groups; normal vs low BMD), we used logistic regression models to verify whether any of these variables were potential confounders. We first fitted an unadjusted crude model with each of the markers followed by models adjusted for age, years since menopause, and BMI. Factors were considered confounders if the change in the odds ratio (OR) as compared with the crude model was >20%.

## Results

Of the 71 subjects, 64.7% had low BMD (L; n = 46). These women were older in age as compared with women having normal BMD (N; n = 25) (*P* = .03); however, there was no significant difference in the number of years since menopause. Weight, height, and BMI were also comparable between the 2 groups (Table 1). Similarly, history of smoking and previous fractures were comparable between the groups (Table 1).

**Table 1.** Demographic data and baseline clinical characteristics of postmenopausal women enrolled in the study (mean ± SD).

	Normal BMD (N; n = 25)	Low BMD (L; n = 46)	*P* (N vs L)	Osteopenia (OSN; n = 31)	*P* (N vs OSN)	Osteoporosis (OSR; n = 15)	*P* (N vs OSR)	*P* (OSN vs OSR)
Age (years)	56.1 ± 5.6	59.6 ± 7.8	**.03**	58.7 ± 7.9	.16	61.3 ± 7.3	**.001**	.14
Weight (kg)	80.5 ± 12.2	75.5 ± 12.7	.13	75.7 ± 12.1	.55	75.2 ± 12.3	.18	.96
Height (m)	158.7 ± 5.2	156.1 ± 6.1	.15	157.3 ± 5.3	.18	153.5 ± 6.9	**.02**	.58
BMI (kg/m^2^)	32.0 ± 5.2	31.0 ± 5.0	.47	30.6 ± 5.0	.28	31.8 ± 5.1	.90	.40
Smoking (%)	2 (8)	2 (4)	.62	2 (6.5)	1.00	0 (0)	.52	.55
Previous fracture (%)	4 (16)	10 (21.7)	.55	7 (22.6)	.62	3 (20)	.69	1.00
Years since menopause (years)	7.6 ± 5.5	9.0 ± 7.2	.53	7.4 ± 6.4	.75	12.1 ± 7.7	**.06**	**.019**
*T*-score hip	0.2 ± 0.8	−1.4 ± 0.87	**.0001**	−1.1 ± 0.8	**.0001**	−2.0 ± 0.7	**.0001**	**.001**
Hip BMC (g/cm^2^)	1.02 ± 0.12	0.78 ± 0.12	**.0001**	0.81 ± 0.13	**.0001**	0.73 ± 0.1	**.0001**	.074
*T*-score L1-L4	−0.11 ± 0.65	−2 ± 0.65	**.0001**	−1.7 ± 0.4	**.0001**	−2.7 ± 0.5	**.0001**	**.0001**
L1-L4 BMC (g/cm^2^)	1.17 ± 0.1	0.90 ± 0.1	**.0001**	0.93 ± 0.09	**.0001**	0.83 ± 0.9	**.0001**	**.003**

BMC, bone mineral content; BMD, bone mineral density; BMI, body mass index.

Significant *P*-values are depicted in bold.

Whereas women with normal BMD (N; n = 25) and osteopenia (OSN; n = 31) had comparable age and years since menopause, osteoporotic women (OSR; n = 15) were significantly older than women with normal BMD (*P* = .001) and had a longer duration since menopause as compared with women having normal and osteopenic BMD (.06 and .019, respectively). All groups had comparable weight, height, and BMI (Table 1).

### Serum levels of bone markers (RANKL, OPG, RANKL/OPG ratio) in serum samples of postmenopausal women with normal or low BMD

As compared with women having normal BMD, women with low BMD had a statistically significantly lower median serum level of OPG (*P* = .004). Although the levels of RANKL were not different between the 2 groups, the RANKL/OPG ratio was significantly higher in women with low BMD (*P* = .027; Table 2).

**Table 2.** Median serum levels of RANKL, OPG, and RANKL/OPG ratio in all groups.

Analyte (pg/mL)	Normal BMD (N; n = 25)		Low BMD (L; n = 46)		*P* (N vs L)	Osteopenia (OSN; n = 31)		*P* (N vs OSN)	Osteoporosis (OSR; n = 15)		*P* (N vs OSR)	*P* (OSN vs OSR)
	Median	IQR	Median	IQR		Median	IQR		Median	IQR		
RANKL	42.4	29.7	42.7	57.9	.721	42.7	65.0	.974	34.0	36.5	.445	.606
OPG	563.3	273.1	468.0	136.4	**.004**	471.3	133.1	**.008**	466.5	164.6	**.025**	.634
RANKL/OPG ratio	0.079	0.06	0.1	0.13	**.027**	0.1	0.17	**.026**	0.88	0.12	.19	.699

BMD, bone mineral density; IQR, intraquartile range; OPG, osteoprotegerin; RANKL, receptor activator of nuclear factor κB ligand.

Significant *P*-values are depicted in bold.

Similarly, as compared with women having normal BMD, both osteopenic and osteoporotic women had lower levels of OPG (*P* = .008 and .025, respectively). Although the differences in serum levels of RANKL did not reach statistical significance between any of the groups, the RANKL/OPG ratio was statistically significantly higher in women with osteopenia as compared with those having normal BMD (*P* = .026; Table 2).

Although the serum levels of RANKL and OPG did not significantly correlate with BMD of the hip and spine, the RANKL/OPG ratio showed weak negative significant correlation with spine BMD (*r* = –0.29, *P* = .046).

### Serum levels of OS markers in serum samples of postmenopausal women with normal or low BMD

**Figure 1.**
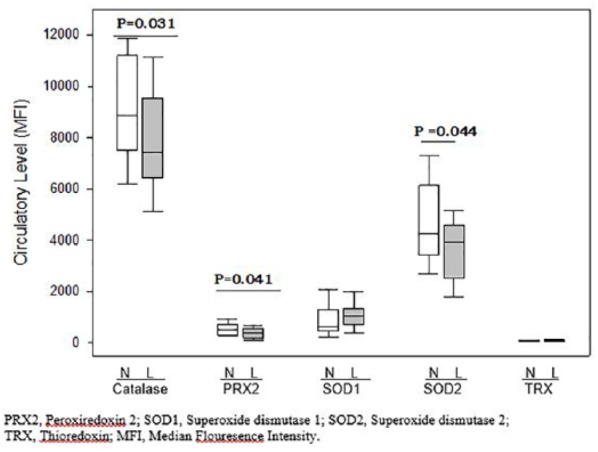
Median serum levels of oxidative stress markers in postmenopausal women with normal (N) and low BMD (L). BMD, bone mineral density.

Serum levels of catalase, SOD2, and PRX2 were significantly lower in postmenopausal women with low BMD as compared with women having normal BMD (*P* = .031, .044, and .041, respectively). However, levels of SOD1 and TRX1 were not significantly different between the 2 groups (Figure 1).

As compared with women having normal BMD, osteopenic women had lower levels of catalase, SOD2, and PRX2 (*P* = .032, .024, and .033, respectively). On the other hand, as compared with women having normal BMD, serum levels of OS markers in osteoporotic women were not significantly different (data not shown).

None of the OS markers showed significant correlation with BMD of the hip or spine.

#### Regression analysis of bone and OS markers and BMD groups

Regression analysis of bone and OS markers and BMD groups in unadjusted and adjusted models for age, years since menopause, and BMI showed that the serum levels of bone markers (OPG and RANKL/OPG ratio) as well as OS markers (catalase, SOD2, and PRX2) were affected by age and years since menopause, indicating that these are confounding factors. However, the same parameters were not affected by BMI.

## Discussion

Numerous studies have focused on the potential association between the serum levels of RANKL, OPG, OS markers, and bone density, but several discrepancies are evident.^9,10,16,21,24^

We report here that circulatory serum levels of OPG in postmenopausal women with normal BMD were significantly higher than those in women with low BMD. On the other hand, although we did not find a significant difference in serum levels of RANKL, women with low BMD had significantly higher RANKL/OPG ratios as compared with women having normal BMD. Our results suggest that the raised levels of OPG are protective, whereas the higher RANKL/OPG ratios may indicate a higher bone turnover and may be associated with lower BMD.

A number of other studies have found varied associations. Some have demonstrated OPG and RANKL to be independently associated with osteoporosis,^28–30^ whereas others have reported OPG-positive, RANKL-negative association with BMD.^31^ The relative expression of RANKL and OPG is reported to be critical in bone remodeling.^10,14–16,32^

In contrast, several studies have not shown any association between BMD and serum levels of OPG or RANKL.^10,32,33^ One study concluded that there was no difference in levels of RANKL between premenopausal women, untreated postmenopausal women, and postmenopausal women on estrogen replacement therapy.^9^ Furthermore, Liu et al^32^ found no differences in serum levels of OPG and RANKL as well as the RANKL/OPG ratio among normal, osteopenic, and osteoporotic women. The role of OPG/RANKL system has also been debated in secondary osteoporosis, such as hepatic osteodystrophy.^34,35^ It has been suggested that variations in circulatory levels of OPG and RANKL may reflect a compensatory reaction to enhanced osteoclastic activity or a result of other inflammatory processes.^34^

Bisphosphonate therapy, in general, is reported to selectively suppress osteoclast activity and thereby retarding bone resorption.^36^ Although it has been widely used in the clinical treatment of several bone resorption diseases including postmenopausal osteoporosis, its precise mechanism of action is not fully elucidated.^36,37^ Whereas some reports show that the inhibitory action of bisphosphonates on bone resorption does not involve the regulation of expression of RANKL and OPG,^37^ others have reported an enhanced expression of RANKL/OPG genes.^38,39^

The inconsistencies in study outcomes may be attributed to a wide range of factors that may influence the variability of these measurements. Some may be due to differences in study design, methodology, and other unknown factors (reviewed in Rogers and Eastell^13^). For example, whereas some manufacturers claim that OPG and RANKL are stable if stored at −20°C, others reported that the storage of samples for more than 6 months at −70°C leads to significant reduction in measurable levels.^13,40,41^ Assay performance, precision, and other pre-analytical factors such as circadian rhythm and exercise effects may also contribute to the variability.^13^ Although this inconsistency will question the clinical utility of serum OPG and RANKL as potential markers of disease activity, it encourages further investigations, identification of the sources of variability, and development of new testing assays.

Other researchers questioned whether the serum levels of OPG and RANKL would reflect the activity of these cytokines in the bone microenvironment and further recommend the measurement of these molecules in tissues. Eghbali-Fatourechi et al^9^ reported a significantly higher expression of RANKL on the surface of T- and B-lymphocytes and bone marrow mononuclear cells from early postmenopausal women as compared with premenopausal or estrogen-treated women. Similarly, Abdallah et al^14^ demonstrated an increased mRNA ratio of RANKL/OPG in bone biopsies from women with hip fractures. However, given the fact that cytokines generally function as part of a large and complex network of other cytokines, chemokines, receptors, and antagonists, it would be logical to analyze simultaneously a wider panel of potential factors.^5,8,42,43^

Several studies have investigated the association between OS index and osteoporotic status.^19,21–24^ OS was further shown to be a powerful stimulant for the increased expression of the proresorptive cytokines such as IL-1, TNF-α, and IL-6, thus inducing osteoporosis.^6,24^ The postmenopausal female population is regarded as being even more vulnerable to OS not only because of old age but also by the lower level of 17β-estradiol (E2), which has been shown to act as an antioxidant.^44^

A wide range of OS biomarkers and laboratory techniques are available, each with its own strengths and limitations.^45^ ROS are usually highly reactive and unstable and have a very short half-life, making them difficult to measure directly. Thus, OS is assessed indirectly by measuring the antioxidant enzyme activity. The 3 main enzyme groups responsible for the control of ROS are SODs, catalases, and peroxidases.^46,47^ Other researchers have also assessed other antioxidant levels such as vitamins E, C, A, B6 and folate.^46^ It is worth mentioning that, as of now, there is no consensus on the most appropriate biomarkers of OS; furthermore, the validity of many of the biomarkers currently in use needs to be confirmed.

Sendur et al^48^ reported a negative correlation between plasma lipid oxidation and BMD values in osteoporotic postmenopausal women compared with healthy subjects. Catalase and glutathione peroxidase are the major antioxidant enzymes that detoxify hydrogen peroxide. Catalase and glutathione peroxidase activities were found to be decreased in postmenopausal osteoporotic women.^49,50^ In addition, plasma levels and activity of SOD and were also reported to be negatively associated with lumbar BMD in humans.^49,51,52^ These data and many others point to the possible adverse effects of OS on bone health. Accordingly, it would be tempting to propose and explore the potential use of antioxidants for the management of age-related bone loss.

There is a growing body of evidence that antioxidants may play a role in preventing osteoporosis. Several studies reported positive effects of different antioxidants (eg, vitamins A, C, and E, selenium, carotenoid, dietary pattern, and many others) on OS parameters, and the levels of bone turnover markers.^53–57^ Whereas some believe that antioxidants should be considered in designing therapeutic protocols, others call for further research to better understand the role of antioxidants in the regulation of bone mass.^55,57^

In this study, we report that the serum levels of catalase, SOD2, and PRX2 are significantly lower in postmenopausal women with low BMD as compared with women having normal BMD supporting the proposed protective function of these enzymes. It is also interesting that women with osteopenia had lower levels of catalase, SOD2, and PRX2 than those with osteoporosis. We propose that, from an immunologic perspective, osteoporosis is the state of progression from osteopenia and is not an immunologically independent condition. The differences in levels of these markers may reflect a compensatory reaction to enhanced osteoclastic activity.^34^

It is interesting that the circulatory levels of RANKL, OPG, and OS markers did not correlate with BMD. Other studies reported similar findings indicating that this bone loss is multifactorial.^10,13,29,30,58^

Whereas studies reporting the association between estrogen levels and bone loss are abundant, epidemiological studies have shown that bone loss starts as early as in the early 30s and long before changes in sex steroid levels.^59,60^ It is now becoming clear that estrogen deficiency by itself is not sufficient to completely explain the pathogenesis of osteoporosis. Several researchers suggest that bone loss is also due to several age-related factors which, in addition to changes in the ovaries, adrenal gland, and kidney, is also due to other factors such as age-related OS, genetic predisposing factors as well as immune and inflammatory mediators.^49,50,59,61,62^ Aging has been characterized by low systemic inflammatory status termed as “inflamm-aging” that may contribute to cytokine and immune balance resulting in proresorptive bias.^6,62^ Collectively, the above lines of evidence encourage moving the pathogenesis of osteoporosis from being estrogen centric to be more multifactorial.^59^

We realize that this study has several limitations. Although measuring the levels of relevant molecules in the blood is less invasive and practical, circulating levels may not reflect the actual levels in tissues. Furthermore, given that our dataset is based on only 71 women and that the data have several variables, the findings and associations should be taken as tentative results, subject to future corroboration. Also, the data presented here do not prove causation. Despite these caveats, our analysis and findings do point to several interesting avenues to be explored further. This may include, but not limited to, circulatory levels of proresorptive/antiresorptive cytokines, bone formation, and degradation markers.

In conclusion, our data provide insights into the possible roles of RANKL, OPG, and OS markers on the pathogenesis of postmenopausal osteoporosis. These data may support future strategies for the prevention and/or reversal of systemic bone loss associated with osteoporosis, where the control of such markers may have therapeutic value.
